# A Novel Foodborne Illness Detection and Web Application Tool Based on Social Media

**DOI:** 10.3390/foods12142769

**Published:** 2023-07-20

**Authors:** Dandan Tao, Ruofan Hu, Dongyu Zhang, Jasmine Laber, Anne Lapsley, Timothy Kwan, Liam Rathke, Elke Rundensteiner, Hao Feng

**Affiliations:** 1 Vanke School of Public Health, Tsinghua University, Beijing 100084, China; dandantao@mail.tsinghua.edu.cn; 2 Data Science Program, Worcester Polytechnic Institute, Worcester, MA 01609, USA; rhu@wpi.edu (R.H.);; 3 Department of Computer Science, Worcester Polytechnic Institute, Worcester, MA 01609, USA; 4 College of Agricultural & Environmental Sciences, North Carolina A & T State University, Greensboro, NC 27411, USA

**Keywords:** social media data, text mining, machine learning, bid data, foodborne diseases, Twitter, PostgreSQL, foodborne outbreaks

## Abstract

Foodborne diseases and outbreaks are significant threats to public health, resulting in millions of illnesses and deaths worldwide each year. Traditional foodborne disease surveillance systems rely on data from healthcare facilities, laboratories, and government agencies to monitor and control outbreaks. Recently, there is a growing recognition of the potential value of incorporating social media data into surveillance systems. This paper explores the use of social media data as an alternative surveillance tool for foodborne diseases by collecting large-scale Twitter data, building food safety data storage models, and developing a novel frontend foodborne illness surveillance system. Descriptive and predictive analyses of the collected data were conducted in comparison with ground truth data reported by the U.S. Centers for Disease Control and Prevention (CDC). The results indicate that the most implicated food categories and the distributions from both Twitter and the CDC were similar. The system developed with Twitter data could complement traditional foodborne disease surveillance systems by providing near-real-time information on foodborne illnesses, implicated foods, symptoms, locations, and other information critical for detecting a potential foodborne outbreak.

## 1. Introduction

Foodborne diseases, arising from the consumption of contaminated food, pose a significant public health concern and have a severe impact on human well-being. Annually, these diseases contribute to a staggering number of illnesses worldwide, leading to 600 million cases and 420,000 deaths [1,2]. Foodborne disease surveillance plays a crucial role in safeguarding human health by monitoring and controlling foodborne diseases and identifying potential foodborne threats. Traditionally, foodborne disease surveillance has relied on data from various sources, such as healthcare facilities, laboratories, and government agencies. The CDC’s National Outbreak Reporting System (NORS), Foodborne Disease Active Surveillance Network (FoodNet), and PulseNet are several foodborne disease surveillance tools used in the United States [3,4,5]. For instance, NORS plays a crucial role in detecting and responding to public health threats by monitoring and reporting foodborne outbreaks in a timely manner. Local health departments report individual cases or clusters of illnesses to NORS, providing demographic information, symptoms, onset dates, exposure history, laboratory results, and other relevant details. Then, NORS integrates data from various sources, performs data analysis, and generates outbreak reports summarizing the findings, including the implicated pathogens, affected population, geographic distribution, potential sources of contamination, and recommended control measures. NORS has been utilized as an important data source in the United States for evaluating the impact of foodborne outbreaks and the associated risk factors with both statistical analysis and data mining methods [6,7,8]. 

Recently, with the advent of social media and the widespread use of online platforms, there is a growing recognition of the potential value of incorporating social media data into foodborne disease surveillance systems [9,10,11,12,13,14]. Social media platforms, such as Twitter, Facebook, and Instagram, have become popular channels for individuals to share their thoughts, experiences, and daily activities. These platforms have also become spaces where users express their health concerns, seek health-related information, and discuss experiences with diseases and outbreaks [10]. As a result, social media data offer a unique opportunity to tap into the collective wisdom and sentiments of the public, providing valuable insights into public health trends, behaviors, and perceptions. For example, Twitter and Yelp were utilized as tools for detecting unreported cases of foodborne illnesses in various local public health departments across the United States. These methods were implemented and evaluated in cities including Chicago, New York, and Las Vegas [11,12,13,14].

However, analyzing social media data poses significant challenges due to their inherent noise, ambiguity, and unstructured nature. Social media platforms provide users with the freedom to express themselves in an unrestricted manner. Consequently, the data generated on these platforms often contain informal language, abbreviations, slang, misspellings, grammatical errors, and sentiment-laden expressions [15]. This “noisy” nature of social media data makes it challenging to extract meaningful insights and gain an accurate understanding from the vast volumes of information available. The analysis of unstructured social media data necessitates the application of advanced natural language processing (NLP) technologies. NLP encompasses a set of computational techniques that enable machines to understand, interpret, and generate human language. It aims to bridge the gap between human communication and machine understanding, facilitating the extraction of valuable information from unstructured text. NLP technologies have made significant advancements in addressing the challenges associated with noisy social media data. These technologies encompass a wide range of techniques, including text preprocessing, tokenization, part-of-speech tagging, named entity recognition, sentiment analysis, topic modeling, and language generation [16]. These advancements have enabled researchers and practitioners to extract meaningful patterns, sentiments, and insights from social media data, supporting various applications such as opinion mining, trend analysis, social network analysis, and public sentiment tracking. In the field of public health, typical NLP techniques have been widely employed to identify potential cases of public threats such as COVID-19 diseases [17]. More recently, the language model BERTweet, a variant of BERT (Bidirectional Encoder Representations from Transformers) was trained to classify relevant Foodborne illness cases from Twitter data [9]. Also, state-of-the-art single- and multi-task deep learning models such as RoBERTa and BiLSTM have been trained to extract critical entities related to foodborne illnesses on Twitter data [18].

This paper aims to explore the potential use of social media data in developing an alternative foodborne disease surveillance system. We discuss the methodologies, tools, and techniques employed in leveraging Twitter data and NORS data for detecting and evaluating foodborne illnesses in the United States, including natural language processing, machine learning, database system, and website development. Furthermore, we address the challenges associated with the use of social media data. By examining the current surveillance system and the potential of social media, this paper highlights the opportunities for developing a complementary and enhanced foodborne disease surveillance system that can leverage the real-time and wide-reaching nature of social media data. One practical advantage of such a system would be the substantial reduction in labor required from experts in the field as they would no longer need to manually examine and extract essential information from social media. Instead, the system could automatically transform the unstructured social media data into a more organized format, focusing on important entities crucial for identifying potential outbreaks of foodborne illnesses.

Overall, the major contributions of this work include the following:

1. We collected a large volume of Twitter data related to foodborne illness and transformed them into a more structured dataset with critical 3W information (PostgreSQL) using the pretrained machine learning models.
2. We compared the descriptive statistics of foodborne illness cases from Twitter data and official NORS data in multiple aspects (numbers, place, time, and food) and built predictive models for predicting cases based on Twitter and/or NORS data.
3. We developed the frontend applications based on the two sources of data for assisting the early detection of foodborne outbreaks.

## 2. Related Work

Foodborne diseases pose significant risks to public health, necessitating effective surveillance systems for early detection, rapid response, and prevention. Traditional foodborne disease surveillance systems primarily rely on data from healthcare facilities, laboratories, and government agencies. However, these systems face challenges such as underreporting, time delays, and limited coverage. The Internet serves as a valuable data source for disease surveillance, enabling the early detection of food safety and food fraud hazards and more digitalized supply chain management in the food industry [19,20]. Across the globe, various information systems have been developed to leverage internet data retrieval and text mining techniques, aiming to enhance early warning capabilities. For example, a Japanese group constructed a database of food safety documents by conducting keyword searches on Google web pages [21]. In Singapore, the National Environment Agency collaborated with IBM Research to establish the Food Safety Information System (FoodSIS), which proactively monitors emerging food safety issues by extracting relevant content from the Internet [22]. In China, a database system of food safety information was created in 2016, utilizing food safety news from media and government websites to facilitate the efficient assessment of food safety concerns [23]. Additionally, a food fraud reporting system MeDISys-FF was developed based on an infrastructure MeDISys that gathers worldwide reports published in the media [24]. In addition to utilizing online Internet information, applications that allow the positive reporting of unpleasant dining experiences can provide another means to record food safety issues. For example, iwaspoisoned.com is an online platform where individuals can voluntarily report incidents of foodborne illnesses they have experienced. Users can provide details about the location, date, symptoms, and the suspected food establishment. The platform aggregates and analyzes these data, providing insights into detecting potential foodborne outbreaks [25]. Database data and text-based data reported in more structured formats are major data sources of these related foodborne illness surveillance systems. 

In recent years, the widespread use of social media platforms has provided an opportunity to explore alternative approaches to foodborne disease surveillance. Social media data are notorious for their unstructured characteristics and for being difficult to analyze, and they have been widely recognized as a potential data source for the early detection of public health threats [10]. In the food safety field, classification models were typically used to identify relevant foodborne illness incidents from Twitter posts [11,12,13,14]. These studies present basic NLP methods and evidence showing that Twitter can provide additional insights into detecting foodborne illness cases in a sentence classification perspective. In addition to sentence-level classification to detect if a tweet indicates a foodborne illness, our previous work constructed token-level models to extract valuable information from Twitter with high accuracy [18]. The availability of what, where, and when (3W) information about people’s everyday lives on social media websites has proven to be valuable for predicting the flu well before outbreaks formally have been reported by the CDC and for preventing public health crises [26]. In the food safety scenario, what refers to the content of the tweet describing a potential food safety incident, e.g., the food product and the complaints about it, while where and when encode the geolocation and the timeframe of the incidents, respectively. Therefore, critical entities such as food and symptoms related to a food safety incident and the location of the incidents are valuable information for detecting a potential foodborne outbreak and, thus, should not be ignored in the full use of Twitter data. The potential value of social media data food safety surveillance has been explored in many previous studies [10,11,12,13,14,15]. Detecting foodborne illness cases via sentence-level text classification models and further examinations of whether positive-predicted incidences can indicate foodborne outbreaks with the assistance of epidemiologists are the major focuses of these work. In contrast, our work, for the first time, attempts to develop a system that can automatically transform the unstructured social media data to a more structured format with 3W information such as food, symptoms, and location, which is essential for the examination of its relevance to foodborne outbreaks. 

## 3. Materials and Methods

### 3.1. Data Collection and the Pipeline

**Figure 1:**
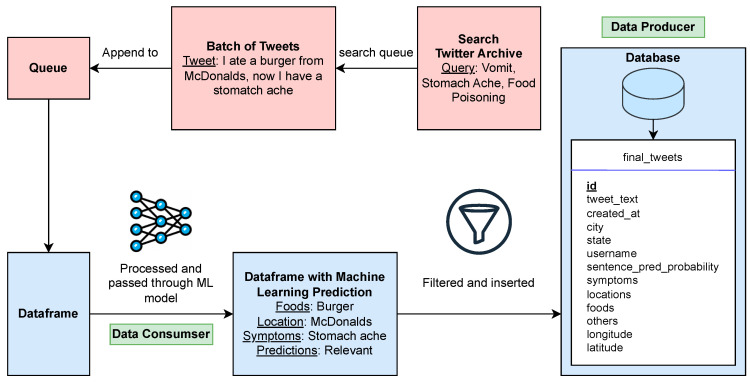
Diagram of the pipeline for Twitter data collection and storage.

Twitter allows researchers who are interested in studying historical tweets to visit its full archive data using Twitter academic accounts. A pipeline (Figure 1) was created to perform the following tasks in sequence. First, to extract the tweets most likely related to foodborne illness, the Tweetkit Python package was used to create a specific query to run on the Twitter archive. The Tweetkit package was used to fit it into our pipeline and was customized to collect all the data associated with a tweet [27]. The pipeline efficiently retrieves tweets from the Twitter archive that may indicate a case of foodborne illness with keywords query: ‘#foodpoisoning’, ‘#stomachache’, ‘food poison’, ‘food poisoning’, ‘stomachache’, ‘vomit’, ‘puke’, ‘diarrhea’, ‘the runs’, ‘nausea’, ‘stomach cramp’, and ‘nauseous’. An automatic system was set up to collect the tweets cyclically in 24 h intervals. Appropriate data cleaning was conducted before data were inserted into the PostgreSQL database. Specifically, only tweets in English were retained for further processing. Duplicates, retweets, and tweets with less than four tokens were removed. Then, the tweets were normalized by converting user mentions and URL links to @USER and HTTPURL, respectively. Since emojis may carry important information, we keep emojis and translate the icons into text strings. These tweets are passed through a refined BERTweet machine learning model, which will provide a prediction on whether the tweet indicates a foodborne illness case. 

Finally, the tweets and their predictions are then fed into the PostgreSQL database with a simple schema to store raw tweets and the attributes, including symptom and food entities extracted by the machine learning model. Tweets related to foodborne illnesses from 2017 to 2021 and with geolocations in the U.S. were collected and stored in the database. The ground truth data of real-world foodborne outbreaks collected from the National Outbreak Reporting System (NORS, https://www.cdc.gov/nors/index.html, accessed on 24 June 2023) were employed to see if the trends observed in the Twitter data correlate to real-world data. The data that are available for each outbreak include year, month, state, primary mode, etiology, serotype or genotype, etiology status, setting, illnesses, hospitalizations, and deaths. 

### 3.2. Information Extraction and Language Models

Twitter provides a unique opportunity to monitor food-safety-related incidents and the temporal and spatial patterns in a near-real-time fashion. Besides time, location, and information embedded in the structured meta data, other valuable information may be expressed in the unstructured text such as the name of the restaurant, grocery store, or dish. In recent years, deep learning models, especially pretrained BERT [28] models, are promising techniques for sequence tagging and named entity recognition (NER) tasks due to their ability to learn from the context surrounding the words in a sequence. In our previous study, single- and multi-task deep learning models including BERTweet [29], RoBERTa [30], BiLSTM [31], and MGADE [32] were trained to extract the critical entities related to foodborne illnesses on Twitter data [18]. As shown in Table 1, important 3W information, such as food, location, and symptoms, that was useful for the analysis of a foodborne illness was extracted by the deep learning models and inserted in the database. The 3W properties together form the core entities essential for food safety outbreak monitoring and prevention. However, with social media data being big data, meaning a high volume of data arriving with high velocity, it is not realistic to conduct reliable analysis on the fly. Instead, we propose to merge the 3W information extracted from external sources into a unified data manager.

### 3.3. Data Storage

A PostgreSQL database was designed to store a large amount of data that could be easily and quickly accessed and queried for our frontend visualizations. The PostgreSQL database allows to store complex data types such as the array data type, which is necessary to store the ‘symptoms’, ‘locations’, ‘foods’, and ‘others’ values as these all contain multiple entity words per tweet. All the collected data were stored in one table named “final_tweets”. This table contains 13 fields, each of which we consider useful for querying on our frontend. For each of these fields, the appropriate data type was selected so that it would be most efficient for frontend use, as well as database storage. The ‘id’ is the primary key of our final_tweets table as it uniquely identifies each individual tweet. Querying the count of ids can be used to obtain tweet counts based on different factors. The ‘id’ column was stored as the bigint type as the integer type is ideal for storing ids in a database. The ‘tweet_text’ column is stored as the text type as we found issues trying to limit storage to a certain number of characters. The ‘created_at’ column stores tweet timestamps as PostgreSQL timestamps so that we can easily query data over certain time intervals. The ‘city’, ‘state’, and ‘username’ columns are all of the varchar type to ensure that these values will be stored without taking up too much space in our database. The ‘sentence_pred_prob’ is stored as a numeric type as it has many digits past the decimal point that we want to preserve for precision. As mentioned above, the ‘foods’, ‘symptoms’, ‘locations’, and ‘others’ entities were stored as text arrays. Lastly, ‘longitude’ and ‘latitude’ were both stored as numeric types in order to account for precision. These 13 fields were retained in our database in order to optimize queries for our frontend. The columns here contain data that are needed to display frontend visualizations or conduct simple statistics. Before inserting the collected data into the database for frontend use, the data were filtered through the pretrained machine learning model so that the tweets having a sentence prediction equal to 1/indicating foodborne illness were included in the database used for frontend interface design. In order to perform a later analysis of all the tweets that ran through the collection process, we implemented raw data storage that contains all the fields initially collected from Twitter.

### 3.4. Frontend Website Design

Next.js (Next.js, 2023), a frontend web development framework based on the React (React, 2023) library, was chosen as our frontend web framework. The implementation of Next.js in the system ensures a responsive user interface and boosts system performance [33]. An additional benefit of Next.js is that it leverages the React frontend library with well-supported React resources online. D3.js (D3.js, 2023) was chosen as our visualization library for the frequency graph, which is widely used and considered by many to be an industry standard. Mapbox (Mapbox, 2023), a hosted GIS mapping service, was used to connect the tweets in our database to an interactive Mapbox map, allowing users to interact with the tweets on a US map. Finally, Tailwind CSS (Tailwind CSS, 2023) was chosen as our UI library as it is small and efficient while also providing significant flexibility to developers. 

## 4. Results and Discussion

### 4.1. Statistics of the Collected Data

**Figure 2:**
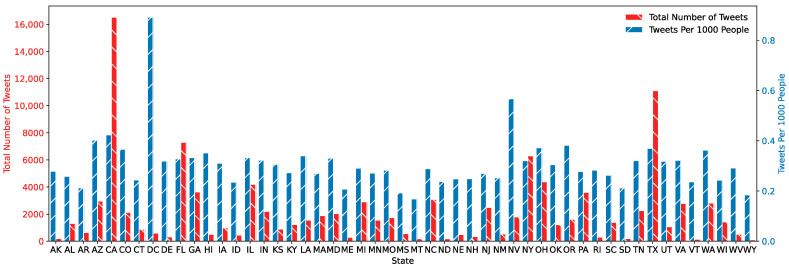
Graph of collected tweets compared to per capita tweets from 2017–2021.

The pipeline collected around 430,000 geolocated tweets from the beginning of 2017 to the end of 2022, with 110,000 predicted as positive for indicating a foodborne illness and stored in the database after passing through a machine learning model. Figure 2 shows the number of tweets by state and the number of tweets per capita. This visualization allows one to visually compare the difference between the number of identified tweets related to foodborne illnesses from each state to the number of tweets by state per capita. 

One potential issue is the repeated author IDs, which could possibly mean a Twitter account dedicated to foodborne illnesses and tweet foodborne illness information rather than instances of foodborne illness, such as news from the CDC, FDA, and associated agencies. This would not be an issue if the tweets were relevant to the research goal, for example, the official Twitter account iWasPoisioned posts about instances of foodborne illness and, thus, should not be excluded from the raw data. Luckily, in searching samples of collected tweets for repeated author IDs, the only one that did appear to come up often was the iWasPoisioned Twitter account, so we deemed that not an issue since the iWasPoisoned Twitter account most likely offers cases of foodborne illness. After filtering the raw data with the pretrained machine learning model, less than 0.5% (less than 300 tweets) of over 56,000 tweets collected from iWasPoisoned were predicted not related to a case of foodborne illness. Meanwhile, the other repeated Twitter accounts had few tweets that were predicted to be related to foodborne illness incidences, posing little impact on the whole dataset.

### 4.2. Descriptive Analysis

**Figure 3:**
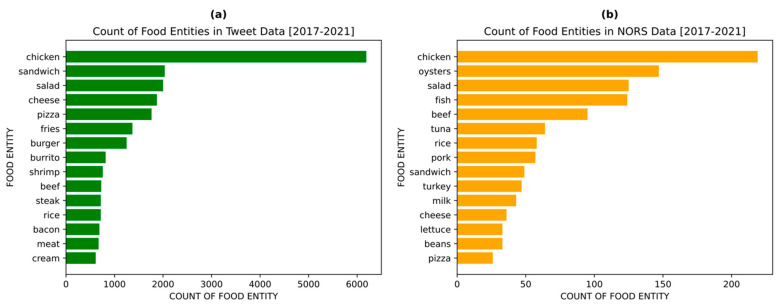
Graph displaying the 15 most frequent food entities found in collected tweets (**a**) and NORS data (**b**) from 2017–2021.

As mentioned above, the pretrained machine learning model extracted ‘food’ entities from a tweet. These entities are identified words that most likely indicate a key food or ingredient. To evaluate the validity of the collected tweets in indicating real foodborne illness cases, the most frequent food entities extracted by the model were compared with the real food vehicles involved in historical foodborne outbreaks reported in the NORS data (Figure 3). As shown in Figure 3a, the top 15 most frequent food entities that appeared in the Twitter database from 2017–2021 were “chicken”, “sandwich”, “salad”, “cheese”, “pizza”, “fries”, “burger”, “burrito”, “shrimp”, “beef”, “steak”, “rice”, “bacon”, “meat”, and “cream”. “Chicken” was identified as the most frequent food entity mentioned in tweets related to foodborne illnesses, with a much greater number compared to the rest of the other food entities. Figure 3b shows the top 15 most frequent food entities mentioned in the NORS reports of foodborne outbreaks from 2017–2021. It was noticed that some of the food entities retrieved from NORS were not necessarily food related. As shown in the figure, “chicken” also appears as the most frequent food entity in the NORS reports. The other frequent food entities are “oysters”, “salad”, “fish”, “beef”, “tuna”, “rice”, “pork”, “sandwich”, “turkey”, “milk”, “cheese”, “lettuce”, “beans”, and “pizza”. Aquatic products appeared more frequently in the NORS reports than in the Twitter dataset. It should be noticed that less frequent food entities such as “lettuce”, “sprouts”, “tomato”, and “lettuce” belong to the category of “vegetable”. Therefore, a comparison of food categories might help to identify patterns in related food entities. 

The Interagency Food Safety Analytics Collaboration (IFSAC) Food Categorization Scheme, used to categorize food sources of contamination in an outbreak and perform attribution analysis, was created by three federal agencies: the CDC, the U.S. Food and Drug Administration (FDA), and the U.S. Department of Agriculture Food Safety and Inspection Services (USDA-FSIS) [34]. Based on the IFSAC category, identified foods are labeled into one of seventeen categories. Identified foods causing historical foodborne outbreaks were labeled with an IAFSC category in the NORS database. Because of the sheer number of unique food entities, grouping the foods into general categories for analysis could provide valuable insight. In order to automatically convert foods extracted from the Twitter data into corresponding categories, we hand-labeled the top 250 common food entities into their respective categories based on the IFSAC Food Categorization Scheme. These labels were put into a JSON dictionary, which could be easily read to convert entities to categories. 

**Figure 4:**
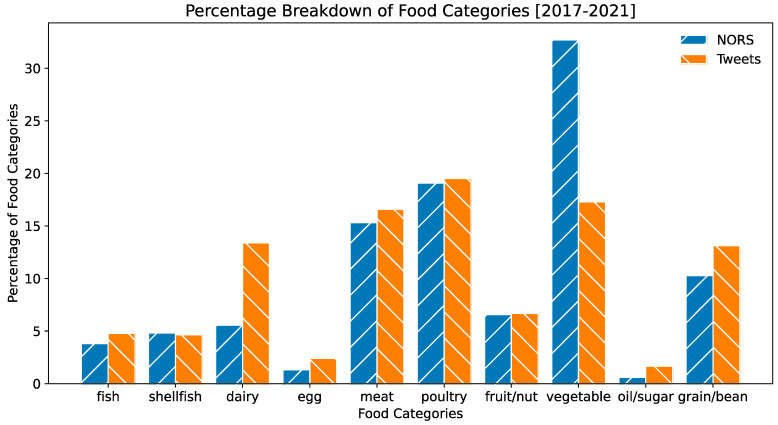
Graph displaying the percentage of food categories in NORS reports and Tweets data.

Figure 4 shows the total percentage breakdown of each category for Twitter data and NORS data. The volume of food categories between the NORS dataset and the Twitter dataset were relatively similar, which is promising for an accurate comparison. The percentage of meat, poultry, fruit/nut, and fish categories in both datasets are close to each other, while NORS reports include a significantly higher percentage of vegetable category than Twitter data. Dairy, oil/sugar, and grain/bean are mentioned more frequently than in the NORS data after the percentage breakdown. Researchers have found similar trends in the percentage composition of food categories in comparison to Yelp data and NORS data collected in 2006–2011 [35]. With a small number of expert evaluation (labeling) data, the pretrained machine learning model combined with an automatic category conversion mechanism found that foods implicated in foodborne illness posts on Twitter correlated with foods implicated in reports from the CDC, indicating that Twitter posts could complement traditional surveillance systems by providing near-real-time information on foodborne illnesses and the implicated foods. 

### 4.3. Outbreak Forecasting Model

Traditional epidemiological models for outbreak forecasting include a variety of regression models, such as timeseries regression, multivariant regression for the prediction of case numbers, and multinomial regression, binary regression, and logistic regression for the prediction of a class, whether it is high/low risk classification or multiple outbreak/sporadic outbreak classification. In the foodborne illness outbreak forecasting scenario, ARIMA and Gaussian distribution models were used to conduct a timeseries analysis of foodborne outbreaks and predict potential outbreaks in India [36]. In addition to timeseries regression models, a multivariate regression model was used for the prediction of cases of Salmonella enterica serovar Enteritidis infections [37]. Regression models can also be regarded as classification models when the goal of prediction turns out to be a class. For example, a multinomial regression model was used to predict caustic pathogens of food poisoning cases for assisting outbreak analysis and forecasting possible pathogens of contamination in future outbreaks [38]. A binary regression model was used to classify the restaurants as high risk or low risk from Twitter data [13]. Logistic regression, a classical statistical regression model in which the response variable is categorical, was employed to classify Yelp reviews indicating “sick” and “multiple outbreaks” [14]. Similarly, logistic regression was used as the classification method in this study with the goal to classify the response variable as “outbreak”–1 or “not outbreak”–0 by establishing a threshold. The threshold is the number of cases in a specific time period, a hyperparameter that could be modified during modeling. This is useful to show days that have more than a certain number of cases, indicating when the higher risk times are. The logistic regression equation resembles the linear regression equation; however, it generates a value ranging from 0 to 1. The predicted label is determined by its proximity to either end of the range.

**Figure 5:**
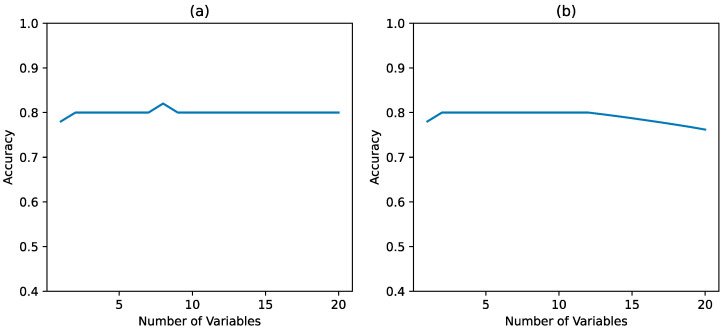
Accuracy of logistic regression with tweets and NORS data (**a**) and with tweets (**b**).

The accuracy score of a logistic model indicates the number of correct predictions made by the model. When Twitter data was used in combination with the NORS data, a best accuracy score of 0.82 was obtained on the validation set (Figure 5a). Since NORS data are not as timely as Twitter Data, we further explored the effectiveness of the models built upon tweets only. For tweets-only modeling, the best accuracy score reached 0.8 on the validation set (Figure 5b), which shows a great chance of employing tweets to detect significant foodborne illness outbreak. In previous studies, classifiers with performance scores of 0.74, 0.84, and 0.64 were obtained with fairly good performance when predicting foodborne illness cases [13,14]. The logistic regression model appears to be quite accurate in predicting whether the NORS and/or Twitter cases will exceed 200. A foodborne outbreak was defined as an event when two or more people get sick after eating the same food [39]. While most of the foodborne outbreaks are sporadic and only affect a small number of people, some outbreaks especially multistate outbreaks often cause a significant number of sicknesses. The prediction model helps to identify time periods with high risk (prediction with the class “outbreak” or “1”) when the number of cases exceeds the threshold. 

### 4.4. Frontend Visualizations

**Figure 6:**
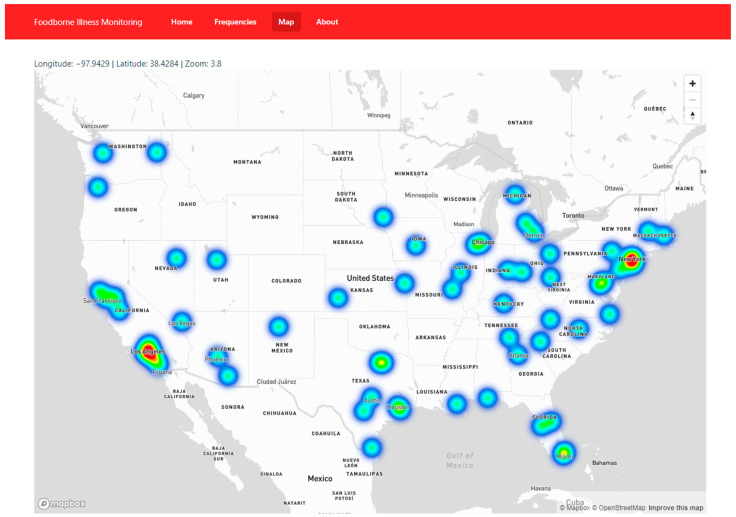
The heat map of tweets within the U.S.

A website was created, and made publicly available at https://usda-foodpoisoning.wpi.edu/, to communicate the results of our work to both policymakers and ordinary people. Three core data sections are included, each presenting a different aspect of our data collection. Firstly, on the homepage, the user is presented with several meaningful statistics about the collected tweets. The number of tweets collected, the number of tweets that we are more than 90% confident were correctly identified by the algorithm, the number of tweets with symptom entities, the number of tweets with city information, and the top five states ranked by tweet volume (California, Texas, Florida, New York, and Ohio) are displayed. The purpose of this section is to provide quick insights into the extensive volume of analyzed tweets. Secondly, a graph is displayed illustrating the top symptoms over time for key foodborne illness-related keywords. This data is generated in real time from the backend since users will have the ability to filter results within a specific time range. An increase in frequency for a particular keyword could indicate the occurrence of a foodborne illness event. Finally, an interactive map (as shown in Figure 6) was created to display the geocoordinates of every tweet containing location data in a heatmap format. Dark shades of red indicate areas with the highest number of tweets about foodborne illness, while teal represents areas with lower foodborne illness tweet density. The heatmap is essential for accurately presenting the data due to a limitation in the geodata received. In most cases, the geolocation provided is a city or neighborhood rather than exact coordinates matching the original user’s location when tweeting. Consequently, multiple tweets often have the same geocoordinates, which are plotted on the map. For instance, there are over 700 tweets with the same coordinates in different parts of New York City. Fortunately, with the properties of the heatmap, an increase in tweets with the same coordinates results in a darker shade of red, enabling users to identify significant tweet volume associated with a specific point.

In addition to zooming in and out, the map supports click actions. When a user taps on a location with tweet density, a popup appears displaying the text of the tweet. This feature is crucial in validating the results of our model as users can independently assess the raw text of a tweet that we have predicted to be related to foodborne illness and form their own conclusion about the accuracy of our prediction. In cases in which multiple tweets are stacked on top of each other, users are provided with “Previous” and “Next” buttons to cycle through potentially hundreds of tweets associated with a specific location.

**Figure 7:**
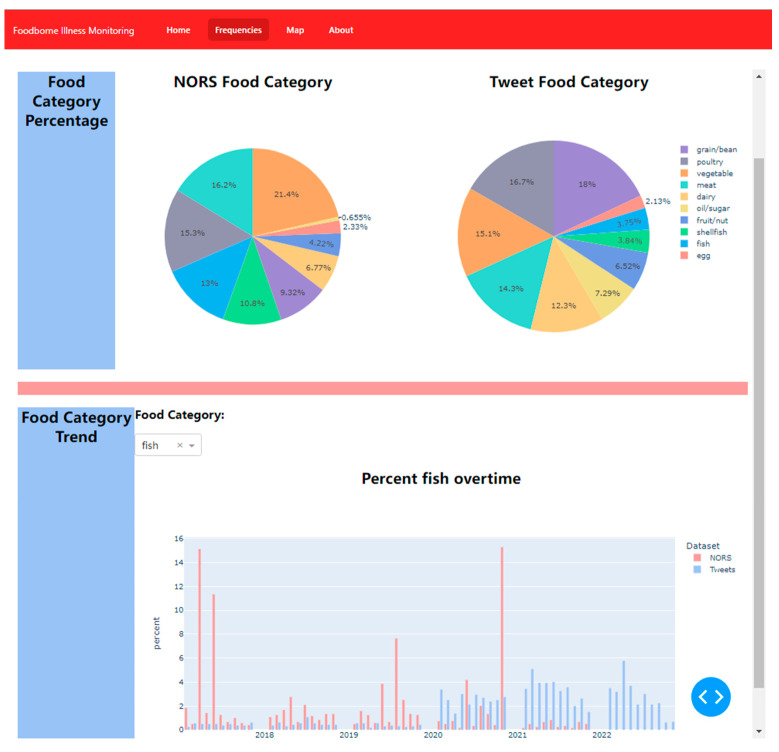
The frequencies of most common food categories related to foodborne illnesses.

The Python module Dash [40] was used to create an interactive dashboard (Figure 7) for the frequency module. An interactive dashboard was developed using the React framework. The focus of this prototype was on interactivity, allowing users to modify date ranges and explore specific food groups. The interactivity of the dashboard can be advantageous for both government officials and the general public, enabling them to gain a broader perspective on potential foodborne illness outbreaks.

### 4.5. Limitations and Implications of This Study

In this study, we successfully developed a pipeline that efficiently collects Twitter data, identifies foodborne illness cases, and extracts important information from those cases. The frontend visualizations also allow users to observe the geographical distribution of potential foodborne illnesses on a map, compare common food categories associated with historical outbreaks (based on CDC data) with the categories detected in positive-predicted cases from Twitter data, and track changes over time for different food categories in both datasets. Overall, our system offers an alternative approach to assist foodborne outbreak surveillance. However, there are certain limitations to consider, as described below.

Firstly, our data collection mechanism relies on sampling Twitter data, which means that a substantial number of foodborne illness cases may be missing from our dataset. Additionally, we only include tweets with geolocation information, which may result in the exclusion of informative tweets lacking such location data. Secondly, when classifying newly captured tweets, we utilize a pre-trained RoBERTa model that demonstrates high accuracy but may have limited generalization capability for unseen tweets. Finally, the existence of multiple platforms can lead to redundancies. For instance, websites like iwaspoisoned.com offer similar functionalities to our system but utilize structured data positively reported by users. In contrast, we aimed to leverage the largely untapped potential of unstructured data from social media, although a portion of our collected data does come from that platform. Despite appearing redundant, these two types of systems can complement each other in various ways, such as evaluating model performance using different datasets and leveraging diverse data sources to assist in identifying foodborne outbreaks.

## 5. Conclusions

A unique information system for monitoring foodborne illnesses was developed by utilizing social media data. By converting unorganized Twitter data into a structured database format that includes essential 3W information, the system enables more efficient detection of potential outbreaks related to foodborne illnesses. One valuable outcome of implementing this system is the reduction in human efforts needed to extract important entities from social media, while also preparing for the analysis of large-scale datasets in close to real time. Through descriptive and predictive analyses of the collected data, we compared the results with the ground truth data reported by the CDC. Our study revealed that the most implicated food categories and their distributions in both the Twitter dataset and NORS dataset were similar. This finding suggests that our system, developed using Twitter data, can complement traditional surveillance systems by providing near-real-time information on foodborne illnesses and implicated foods. The outbreak forecasting model also helps to identify time periods with a high risk of causing a certain number of cases. By incorporating social media data into surveillance systems, we can benefit from timely and comprehensive insights, allowing for quicker responses and more effective control measures. In conclusion, this research highlights the potential of social media as a valuable resource for enhancing foodborne disease surveillance and ultimately improving public health outcomes. The continued exploration and refinement of such alternative surveillance approaches will contribute to the development of more robust and proactive systems for monitoring and preventing foodborne diseases.

## Figures and Tables

**Table 1:** Examples of tweets processed by the language model.

Tweets	Food	Location	Symptom
Biggest regret of #2013: eating sushi from safeway’s refrigerated section #foodpoisoning #projectilevomitingatmidnight	Sushi	Safeway	#projectilevomitingatmidnight
@USER I ate like 3 tacos from jackinbox lastnight in when I woke up my stomach been hurting every since I think I got food poison	Tacos	Jackinbox	Stomach been hurting
@USER @USER Bought family meal in Rustenburg on my way from mahikeng last weekend. All four of us had severe cramps and running stomach the next day. Went to the doctor and was told it’s food poisoning. Never again will I buy nandos HTTPURL	Nando’s meal	Rustenburg	Severe cramps, running stomach
Panda Express got me@and three others sick. Orange chicken.. we all ate different day at different locations!! What the hell is going on? I get my days off till next week. Thanks Panda I am hoping I don’t puke on my staff [EMOJI_disappointed_face][EMOJI_disappointed_face][EMOJI_disappointed_face] #pandaexpress #foodsafety #foodpoisoning	Orange chicken	Panda Express	Sick, puke

## Data Availability

The datasets collected and analyzed during the current study are available from the corresponding author on reasonable request. In addition, we would like to clarify that the terms of service of Twitter were followed in order to collect the data used in our study. The CDC dataset of historical foodborne outbreaks would be available from a CDC National Outbreak Reporting System (NORS) data request application by emailing the NORS Dashboard mailbox.
